# Diagnostic and pathogenetic role of café-au-lait macules in nevoid basal cell carcinoma syndrome

**DOI:** 10.1186/1897-4287-10-15

**Published:** 2012-10-29

**Authors:** Giovanni Ponti, Aldo Tomasi, Lorenza Pastorino, Cristel Ruini, Carmelo Guarneri, Victor Desmond Mandel, Stefania Seidenari, Giovanni Pellacani

**Affiliations:** 1 Department of Clinical and Diagnostic Medicine and Public Health, University Hospital of Modena and Reggio Emilia, University of Modena and Reggio Emilia, via del Pozzo 71, Modena, Italy; 2 Department of Internal Medicine and Medical Specialties (DiMI), University of Genoa, Genoa, Italy; 3 Department of Surgical, Medical, Odontoiatric and Morphological Sciences, with Transplantation, Oncological and Regenerative Medicine interests, Division of Dermatology, University Hospital of Modena and Reggio Emilia, University of Modena and Reggio Emilia, via del Pozzo 71, Modena, Italy

**Keywords:** Café au lait spots, Nevoid basal cell carcinoma syndrome, *PTCH1* mutation, Neurofibromatosis type 1, Genodermatoses, Hereditary cancer syndrome

## Abstract

Café au lait spots (CALS) are common dermatologic findings that can at the same time arise in a variety of pathologic conditions such as Neurofibromatosis type 1 (NF1), together with numerous hereditary syndromes for which they represent either diagnostic criteria or associated elements (McCune Albright, Silver-Russell, LEOPARD, Ataxia-Telangiectasia). A review of the literature also revealed two cases of association with NBCCS. We report here the case of a female proband with CALS associated to Nevoid Basal Cell Carcinoma Syndrome (NBCCS) with known PTCH1 germline mutation (C.1348-2A>G) who had been misdiagnosed with NF1 in her childhood because of 5 CALS and cutaneous nodules. The patient presented a giant cell tumor of the skin, palmar and calcaneal epidermoidal cystic nodules, odontogenic keratocystic tumors and deformity of the jaw profile. Her family history brought both her brother and father to our attention because of the presence of KCOTs diagnosed at early age: after genetic testing, the same PTCH1 germline mutation was identified in the three family members. Clinical criteria are used for discerning NF1 diagnosis (size, number and onset age), while there are no definite guidelines concerning CALS except for their presence. In our experience, we have noted an association of CALS with NBCCS; this seems interesting because we already know clinical criteria are a dynamic entity and can be modified by epidemiologic evidences.

## Background

Café au lait spots (CALS) are cutaneous hyper pigmented flat macules or patches (>1 cm) that usually appear in childhood and tend to increase in number and size until puberty [1]. They’re colored in various shades of brown and located anywhere on the body, independent from sun exposure, especially on face, scalp, palms, soles and external genitalia. Although a single CALS is a common finding in Caucasian children (10-20%) [2], an increasing number is much less frequent: 6 CALS represent a threshold for the diagnosis of Neurofibromatosis type 1 [3,4]. NF1 isn’t the only disease associated to CALS, that appear in multiple pathologic conditions for which they represent either diagnostic criteria or just associated signs: McCune-Albright syndrome, LEOPARD syndrome, Ataxia telangiectasia syndrome and many more (see Table 1) [5]. They’re a common finding of metabolic disease such as Gaucher syndrome and they were also reported in patients in two cases of Nevoid Basal Cell Carcinoma Syndrome (NBCCS).

**Table 1:** Syndromes associated with café-au-lait macules

**Syndrome**	**Gene or Locus**	**Cutaneous Clinical Features**	**Systemic Clinical Features**
NF1	NF1	Multiple café-au-lait (>6), skin-fold freckling, cutaneous and plexiform neurofbromas	Macrocephaly, optic pathway glioma, skeletal dysplasia
NF2	NF2	Café-au-lait macules seen but not a criterion for diagnosis, neurofibromas	Acoustic neuromas, schwannomas, meningiomas, juvenile posterior subcapsular lenticular opacity
Multiple familial Café-au-lait	Unknown	Multiple café-au-lait	Without other stigmata of NF1
Legius (NF-1 like) syndrome	SPREAD1	Multiple café-au-lait, skin-fold freckling	Without other stigmata of NF1
McCune Albright syndrome	GNAS1	Segmental café-au-lait	Precocious puberty, other endocrinopathies, polyostotic fibrous dysplasia
Constitutional MMR deficiency syndrome	MLH1, MSH2, MSH6, PMS2	Multiple café-au-lait	Adenomatous colonic polyps, multiple malignancies (medulloblastoma, lymphoma, glioblastoma)
Ring chromosome syndrome	Choromosomes 7,11,12,15,17	Multiple café-au-lait	Microcephaly, mental retardation, short stature
Leopard/multiple lentigenes syndrome	PTPN11	Café-au-lait, café-noir, lentigines	Cardiac conduction defects, ocular hypertelorism, pulmonary stenosis, growth retardation, hearing loss
Cowden syndrome	PTEN	Café-au-lait spots, Facial trichilemmomas, soft tissue tumors (lipomas, neuromas)	Cobblestoning of the oral mucosa, gastrointestinal polyps, breast carcinoma, thyroid adenoma and cancer
Banayan-Riley- Ruvalcaba syndrome	PTEN	Pigmented genital macules, Facial trichilemmomas	Oral papillomas, gastrointestinal polyps, Macrocephaly, vascular anomalies
**WEAK ASSOCIATION**			
NAME (naevi, atrial mixoma, ephelides) syndrome	Unknown	Naevi, ephelides	Atrial mixoma
Ataxia teleangectasia	ATM	Cutaneous and ocular teleangectasias	Cerebellar ataxia, immunodeficiency, hypogonadism, lymphoreticular malignancy
Epidermal Nevus syndrome	Unknown	Linear epidermal nevus	Mental retardation, seizures, movement disorders
Turner Syndrome	X-chromosomal anomalies (XO karyotupe or Xp deletion)	Cutaneous lymphatic malformations	Short stature, broad chest, low hairline, low-set ears and webbed necks, swelling, gonadal dysfunction, congenital heart disease, hypothyroidism.
Silver-Russel Syndrome	Unknown	Multiple café au lait macules	Short stature, craniofacial and body asymmetry, microcephaly, congenital cardiac defects
Fanconi Anemia	FANCA, FANCB/C/D locus on chromosome 3, FANCE/F/G/H	Hyper- and hypopigmentation of the skin, mucocutaneous squamous cell carcinomas	Bone marrow failure, multiple congenital anomalies, mental retatrdation, microcephaly
Westerhof Syndrome	unknown	Hypopigmented and hyperpigmented macules	Retarded growth and mental deficiency
MEN1/Men2B	RET	Multiple malignancies	
Bloom syndrome	RECQL3	Hypo- and hyper-pigmented spots; telangiectasias	Mental retardation, short stature
Gaucher Disease	Chromosome 1	Yellowish-brown skin pigmentation	Astenia, diarrhoea, ataxia, splenomegalia, hemorrhagies, muscolar atrophia,
Hunter Disease	X-linked	Skin eruptions	Macrocephaly, mental retardation, valvular dysfunction
Watson Syndrome	NF1	Axillary/inguinal freckling	Mental retardation, short stature, pulmonary valvular stenosis, Lisch nodules

## Case presentation

**Figure 1:**
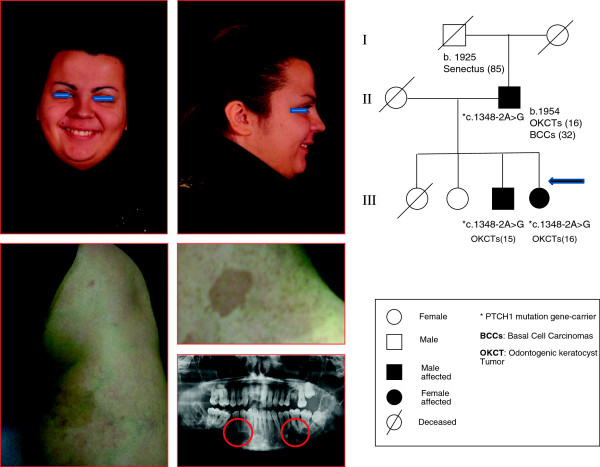
Clinical features and genealogic tree of NBCCS’ probands.

We present here an association between NBCCS and café-au-lait spots, the case of a 23 year old female patient born in 1989, originally examined in the Department of Pediatrics at the age of 10. General and skin examination revealed 4 *café-au-lait spots*, palmar and calcaneal cystic nodules and deformity of the jaw profile (see Figure 1). One of the nodules was excised and histopathological examination set the diagnosis of giant cell tumor. Neurofibromatosis type 1 was the first diagnosis although the patient didn’t completely fulfill the criteria. Later, the patient presented jaw keratocystic odontogenic tumors (KCOTs) that were surgically removed and histologically evaluated. Her family history brought both her brother and father to our attention because of the presence of KCOTs in all of them; they were tested for *PTCH1* gene mutation under suspicion of Gorlin syndrome: diagnosis was made after the discovery of the same *PTCH1* gene germline mutation (C.1348-2A>G). The brother presented KCOTs diagnosed at the age of 15, while the father presented KCOTs diagnosed at the age of 16, so that we hypothesized the presence of a “Gorlin syndrome with KCOTs only”, while a BCC was discovered on the father’s arm was after the dermatologic follow-up to determine whether this was just a sporadic skin tumor or the sign of a full phenotype.

## Conclusion

Nevoid basal cell carcinoma syndrome (NBCC; also known as Gorlin syndrome; OMIM #109400), inherited in an autosomal dominant pattern, is characterized by a very wide spectrum of peculiar clinical manifestations. The most common features include multiple basal cell carcinomas, KCOTs, palmar and/or plantar pits and skeletal abnormalities (i.e. fused, bifid or splayed ribs). According to Kimonis et al., two major or one major and two minor criteria should contemporary exist in order to confirm the diagnosis of NBCCS [6]. Most individuals present developmental defects, such as intracranial calcification, calcifications of the falx cerebri, and a variety of other benign or malignant tumors, including ovarian fibroma, medulloblastoma, rhabdomyosarcomas and cardiac fibromas [7]. The major criteria included multiple BCCs or one BCC before 30 years, keratocysts of the jaw, palmar/plantar pits and lamellar calcification of the falx cerebri on skull radiograph. Minor criteria included spina bifida occulta or other vertebral anomalies, brachymetacarpaly in at least one limb, hypertelorism or telecanthus, frontal bossing, rib anomalies (bifid, synostosed, hypoplastic), ovarian fibroma, medulloblastoma, flame shaped lucencies in the phalanges, and brachymetacarpaly in the 4 limbs. One diagnosis was also established by the presence of a first degree relative with NBCC and one major or two minor criteria. [7].

Our proband meets the diagnostic criteria for Gorlin syndrome since she presents two major criteria: multiple histologically proven odontogenic keratocysts occurred before the age of 20 and family history of NBCCS (father and brother). Moreover, molecular characterization reported the same germline *PTCH1* mutation, C.1348-2A>G [8,9]; we had hypothesized this mutation was related to a NBCCS subset with keratocysts only, until we discovered the presence of one basal cell carcinoma in the proband’s father. The entire family, which has been identified by a clinical approach starting from the KCOTs (8), is still under strict dermatologic follow-up.

We present here the case of an association between NBCCS and café au lait spots; the family history was peculiarly interesting since the proband was initially misdiagnosed with NF1 due the presence of 5 café-au-lait spots, which represent a common dermatologic finding either sporadic or associated to genodermatoses and other hereditary syndromes such as NF1, McCune-Albright syndrome and LEOPARD syndrome.

CALS vary from innocent findings to stigmata that connect different hereditary and sporadic syndromes. Histopathologically, they are nests of pigmented melanocytes, cells of neuroectodermal origin that migrate during the embryonic development: for this reason they sometimes have a somatotopic distribution, sometimes they follow dermatomes [10]. They might for this reason relate to different neoplasms of common embryonic origin, not only in genodermatoses as NF1 but also in many other syndromes. We do not know how frequent is the presence of café-au-lait spots in Gorlin syndrome, but it might be interesting to further analyze this skin feature that might be useful in the detection of NBCCS. The literature review reported the case of a 10 year-old child diagnosed with NBCCS presenting CALS and neck pits [11], and a family composed by women and her two sons with NBCCS and CALS [12]. In general, CALS range from the spectrum of innocent finding to that of an alarm bell for suspecting a hereditary syndrome; the threshold between the two is a clinical criteria, comprehending their number, their size and their onset age, at least in NF1, since in the majority of other syndromes there are no specific guidelines concerning CALS except for their presence. In our experience, we have noted an association of CALS with NBCCS; this seems interesting because we already know clinical criteria are a dynamic entity and can be modified by epidemiologic evidences, as it’s now happening to ameloblastoma in NBCCS diagnosis [13]. Further clinical study will be necessary for the complete characterization of the clinical association between CALS and NBCCS.

## Consent

Peripheral blood samples were collected from the proband and her first-degree relatives. Molecular analysis of *PTCH1* was performed as previously described [14].Written informed consent, agreeing to peripheral blood sampling and genetic analysis, was obtained from each patient. Molecular analysis of *PTCH1* was performed. The *PTCH1* cDNA sequence from GenBank (Accession number U59464.1) was used as a reference sequence, where the A of the ATG translation initiation start site represents nucleotide +1. An Institutional Review Board (IRB) approval was obtained and the study in which the patients were enrolled was conducted according to the Declaration of Helsinki Principles. All patients provided their written informed consent for the management of personal data and for publication of their photographs before participating into the study. A copy of the written consent is available for review on request.

## Abbreviations

BCC: Basal cell carcinoma; CALS: Café-au-lait spots; KCOT: Keratocystic odontogenic tumor; NBCCS: Nevoid basal cell carcinoma syndrome; NF1: Neurofibromatosis type 1.

## Competing interests

The authors declare that they have no competing interests.

## Authors’ contributions

GP: Study concept and design; acquisition of data; drafting of the manuscript. AT: Study supervision. CR: Acquisition of data and Contribution to drafting of the manuscript; CG, VMD: Acquisition of data. LP: Genetic analysis and interpretation of data. SS,GP: Acquisition and interpretation of data. All authors read and approved the final manuscript.
